# Prevalence of Dementia, associated Co-morbidities, and Multidisciplinary Team Involvement in a Psychiatry of Old Age Service

**DOI:** 10.1192/j.eurpsy.2024.1320

**Published:** 2024-08-27

**Authors:** S. Crowley, E. Dunne

**Affiliations:** Psychiatry of Old Age, North Lee Mental Health Service, Cork, Ireland

## Abstract

**Introduction:**

Dementia is a common diagnosis in service users seen by Psychiatry of Old Age (POA) Services. This clinical audit was conducted prior to the services engagement with a focus group, which aimed to explore the implementation of the “Appropriate prescribing of psychotropic medication for non-cognitive symptoms in people with dementia” (National Clinical Guideline No. 21) and identify additional resource requirements to be submitted for consideration by the HSE’s estimate process for 2023.

**Objectives:**

Its aims were to evaluate:

- The prevalence of service users with a dementia diagnosis among those seen by the POA Service, from January 2018-June 2022
- The prevalence of co-morbid psychiatric diagnoses among those with a dementia diagnosis.
- The resources needed to manage currently active cases with a diagnosis of dementia, by evaluating MDT member involvement.

**Methods:**

Data is routinely collected on service users treated by the POA service for service evaluation, including service users’ diagnoses, and current MDT member involvement. All service users seen by the POA service between Jan 2018 – June 2022 were included. The total number of service users, and service users with dementia and mild Cognitive impairment were counted, in order to evaluate the prevalence of dementia. We then evaluated the proportion of those with dementia who had co-morbid psychiatric diagnoses. We then looked at currently active cases with dementia, and evaluated how many MDT members were involved in their ongoing care.

**Results:**

392 service users were treated by the service from Jan 2018-June 2022. Of these 104 cases were still active with the service. 152 (39%) of these service users had a diagnosis of dementia. Of those with dementia, 45% (68, n=152) also had another psychiatric co-morbidity. Psychosis was the most common psychiatric co-morbidity, seen in 22% of those with dementia (33, n=152). 12% of active service users with a dementia diagnosis were only seen in outpatients clinics only, 60% were seeing one MDT member, 28% were seeing multiple MDT members (n=25).

**Conclusions:**

Dementia was the most common diagnosis among service users seen by the POA service. 45% of service users with dementia being seen by the POA service also had another psychiatric co-morbidity. Such patients require significant MDT input.

**Disclosure of Interest:**

None Declared

